# Clinical effectiveness of double-layer artificial dermis transplantation repairing hand wounds with limited bone and tendon exposure

**DOI:** 10.12669/pjms.41.8.11679

**Published:** 2025-08

**Authors:** Congqiang Rao, Jiamin Chen, Binggang Xiong, Hanjie Ou

**Affiliations:** 1 Congqiang Rao Burns and Hand-Foot Surgery, Dongguan Songshan Lake Central Hospital, 523326, Dongguan, Guangdong, China; 2 Jiamin Chen Burns and Hand-Foot Surgery, Dongguan Songshan Lake Central Hospital, 523326, Dongguan, Guangdong, China; 3 Binggang Xiong Burns and Hand-Foot Surgery, Dongguan Songshan Lake Central Hospital, 523326, Dongguan, Guangdong, China; 4 Hanjie Ou Burns and Hand-Foot Surgery, Dongguan Songshan Lake Central Hospital, 523326, Dongguan, Guangdong, China

**Keywords:** Bone, Double-layer artificial dermis, Tendon, Wound healing, Scar

## Abstract

**Objective:**

Our objective was to investigate the clinical efficacy of double-layer artificial dermis transplantation in repairing hand wounds with limited bone and tendon exposure.

**Methods:**

This study involved 46 patients with limited bone and tendon exposure in the hand who were admitted to Dongguan Songshan Lake Central Hospital between January 2022 to January 2024. The patients were randomly divided into two groups: observation and control (N=23/group). The observation group underwent double-layer artificial dermis transplantation, whereas the control group received conventional treatment. The two groups were compared in terms of the overall wound condition, infection rate, granulation tissue growth time, wound healing time, pain scores, scar scores, and hand function recovery scores.

**Results:**

In the observation group, wound swelling and exudation were significantly reduced post-treatment, while no wound infections were observed. In contrast, the control group had significantly more severe wound swelling and exudation, with an infection rate of 13.04%. The observation group had shorter average times for granulation tissue growth and wound healing. Pain scores at days 3, 7, and 14, Vancouver Scar Scores (VSS) and Arm, Shoulder, and Hand Disability Scores (DASH) at six months were significantly lower in the observation group (P<0.05).

**Conclusion:**

The double-layer artificial dermis could effectively reduce the wound’s inflammatory response, control infections, alleviate patient pain, accelerate granulation tissue growth and shorten the wound healing time. Furthermore, it could reduce scar tissue formation and restore the hand’s functionality.

## INTRODUCTION

With technological advancements and widespread use of machinery, individuals operating machines have reported increased incidences of soft tissue and skin defects, as well as bone and tendon exposure.^1^ The thin soft tissues of the hand are particularly vulnerable to mechanical injury, which can result in bone and tendon exposure. Flap surgery is the most commonly employed method for treating wounds with bone and tendon exposure.^2^,^3^

However, patients with extensive soft tissue defects, or who are unable to undergo prolonged periods of anaesthesia, may be deemed ineligible for flap surgery.^4^ Moreover, numerous patients decline flap repair treatment due to donor site trauma, among other postoperative complications. Consequently, free skin grafting has become a prevalent conservative treatment for these patients.

However, it should be noted that this procedure is only suitable for wounds with well-grown granulation tissue. Moreover, infection, as well as tendon and bone necrosis are frequently unavoidable during the process. It is also noteworthy that these complications could result in serious scarring in the later stages, affecting both the physical appearance and function of the affected hands. Therefore, the restoration of the normal appearance and function of the affected hands, with minimal trauma, remains a clinical challenge.

Double-layer artificial dermis, a tissue-engineered dermal substitute, has gained significant attraction in the domain of deep wound repair and scar reconstruction, providing substantial therapeutic benefits.^5^-^7^ The composition of this substitute comprises an upper layer consisting of a semi-permeable silicone membrane and a lower layer consisting of a porous matrix. The matrix is constituted by terminated peptide, bovine Achilles tendon collagen and glycosaminoglycans (GAGs). While the dermal layer is a degradable matrix that could provide a scaffold cell and facilitate capillary ingrowth,^8^ Two to three weeks are often required from its application until dermis-like tissue develops.^9^ As demonstrated in numerous reports, the prolonged utilisation of this acellular tissue-engineered skin may potentially elevate the risk of wound infection, particularly in cases of severe, recalcitrant wounds.^10^-^12^

Additionally, the efficacy of tissue-engineered skin in enhancing scar quality during the advanced stages of wound healing, particularly in cases of burns, remains a subject of debate and controversy.^11^,^13^ The recovery of hand injuries directly impact the restoration of hand function and mobility. In severe cases, this may result in long-term disability, potentially leading to unemployability for the patient. Therefore, we sought to evaluate the efficacy of the double-layer artificial dermis method in treating hand wounds with limited bone and tendon exposure.

## METHODS

This study involved 46 patients (30 males and 16 females; aged between 14 and 68 years) with hand wounds (exhibiting limited bone and tendon exposure) who were treated at the Dongguan Songshan Lake Central Hospital between January 2022 to January 2024. The patients were randomly categorised into two groups: observation and control (N=23/group). The two groups showed no significant differences in age, gender, wound area and wound type (P>0.05) (Table-I).

**Table-I:** Comparison of the two groups’ basic data.

Variable	Observation	Control	p
Sex (male/female)	16/7	14/9	0.536
Age, year (mean ± SD)	35.56±10.48	40.65±16.67	0.183
Wound area, cm2 (mean ± SD)	22.99±11.22	20.91±12.45	0.555
Exposed area, cm2 (mean ± SD)	2.70±1.59	2.37±1.40	0.452
Leakage type (Tendon/bone/Both)	23	23	0.492
Tendon	8	9	
Bone	14	11	
Tendon and bone	1	3	

### Ethical approval:

The ethical committee of our hospital approved our study with the number Dongsong Medical Research Lunshen 2024 No. 56 on September 30th 2024.

### Inclusion criteria:

- Patients with various injuries leading to hand wounds featuring exposed bones and tendons (bone and tendon exposure area < 10 cm^2^).
- Patients aged < 80 years.
- Patients with no severe respiratory or circulatory system diseases.
- Patients with no severe wound or systemic infections.
- Patients ineligible for free flap or pedicle flap surgery, or those voluntarily rejecting flap transplantation surgery due to subjective or objective factors.

### Exclusion criteria:

- Patients who cannot adhere to regular follow-ups.
- Patients with severe organic lesions of the heart, liver, kidney, and lung, among other organs.
- Patients with abnormal coagulation function, active infection, or other untreatable serious internal diseases.

### Methods:

### Observation group:

After admission, the patients underwent thorough debridement of necrotic muscles, tendons, and foreign bodies, with a focus on protecting the periosteum and tendon sheath. Wounds were then repeatedly irrigated with physiological saline and subjected to thorough hemostasis. Following this, the double-layer artificial dermis (Lando, Shenzhen Qikang Medical Equipment Co., Ltd.) was soaked in normal saline for three minutes, trimmed to the shape of the wound, applied to the wound and sutured along the edges. In cases of excessive secretion or bleeding, drainage perforations were made using a sharp blade before wrapping the silicone layer with Vaseline gauze to ensure uniform pressure and a balanced fit.

**Fig.1:**
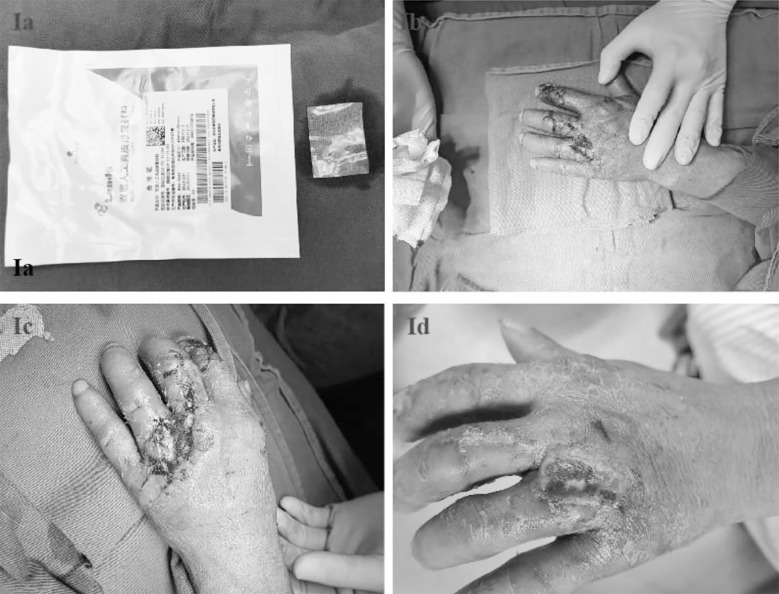
Double-layer artificial dermis and its use Label. Fig.1: (Ia) Double-layer artificial dermis; (Ib) Exposure of tendons on the dorsal side of the left hand; (Ic) Immediately after artificial dermis implantation surgery; (Id) Silicone layer was removed two weeks after double layer artificial dermis implantation;

On the third day post-surgery, the dressing was removed and any fluid or blood accumulated beneath the skin was meticulously drained. Thereafter, the dressing was changed and the process was repeated after every two to three days. The degree of vascularisation of the dermis was observed during dressing changes. Once full dermal vascularization was achieved, free skin grafting surgery was performed, and skin was extracted from the thigh area. Notably, for small wounds (such as nail bed wounds), the artificial dermis could be covered with the epithelium from the edge of the wound, thus obviating the need for the second stage of skin grafting surgery (Fig.1).

### Control group:

After admission, the patients underwent a thorough debridement of necrotic muscles, tendons, and foreign bodies, with a focus on protecting the periosteum and tendon sheath. Wounds were then repeatedly irrigated with physiological saline and subjected to thorough hemostasis. Following that, VSD negative pressure drainage and dressing change therapies were administered. Once the granulation tissue covered the wound completely, free skin grafting was performed. There was no need for second stage skin grafting surgery for small wounds.

### Observation Indicators:

- General condition of the wound: Patients’ wound redness and exudation were recorded post-treatment.
- Pain score: Patients’ pain scores during treatment were recorded using a numerical rating scale.
- Wound infection: The wound exudate was collected for bacterial culture and then the wound infection rate was determined (wound infection rate = number of patients with positive bacterial culture / number of all patients * 100%).
- Granulation tissue growth time: The time when the wound was completely covered with fresh granulation tissue.
- Wound healing time. The time when the wound was completely covered by epithelial cells without any exposed granulation tissue.

Six months after the wound healed, the degree of scarring was evaluated using the Vancouver Scar Scale (VSS). The scale encompasses various parameters including color (total score = 3 points), thickness (total score = 4 points), vascular distribution (total score = 3 points), and softness (total score = 5 points). Lower scores indicated more normal appearances.

Six months after the wound healed, the patients completed self-assessments using the Disabilities of the Arm, Shoulder, and Hand (DASH) scale. The scale comprises 30 items, including functional activities and clinical symptoms, with each item scored using points ranging from 1 to 5. The scales’ total score ranges from 30 to 100, with 30 and 100 being the lowest and highest scores, respectively. Notably, this is a negatively scored scale; hence, the scores of 100 and 30 indicated complete functional impairment and normal functioning, respectively.^14^

### Statistical analyses:

All statistical analyses were performed using the SPSS 22.0 statistical software. Measurement data were expressed as mean ± SD (standard deviation), and comparisons between the two groups was performed using a t-test. Count data were compared using the X^2^ test. Results with P<0.05 were considered statistically significant.

## RESULTS

All 46 cases of hand wounds with bone and tendon exposure healed. In the observation group, 20 cases healed by natural epithelialization and three cases underwent secondary skin grafting. And, in the control group, 18 cases healed by natural epithelialization and five cases underwent secondary skin grafting. The results of the study indicate significant improvements in the observation group. Specifically, the observation group exhibited a significant reduction in swelling and exudation, with no cases of wound infection reported. In stark contrast, the control group experienced more pronounced swelling and exudation, along with a notable infection rate of 13.04%.

**Fig.2:**
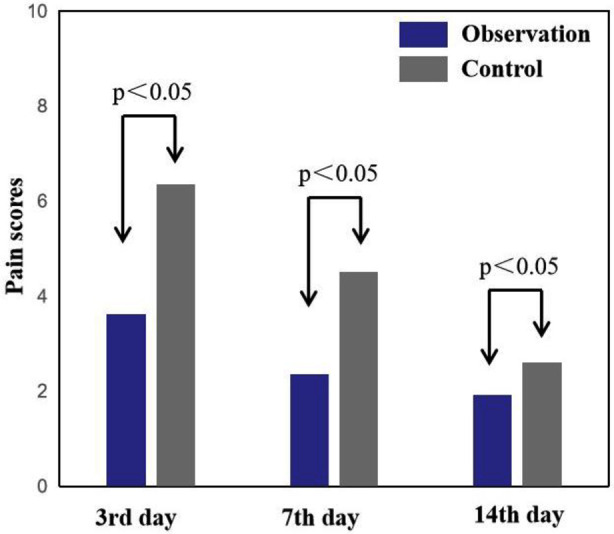
Comparison of the pain scores on days 3, 7, and 14 between the two group.

**Fig.3:**
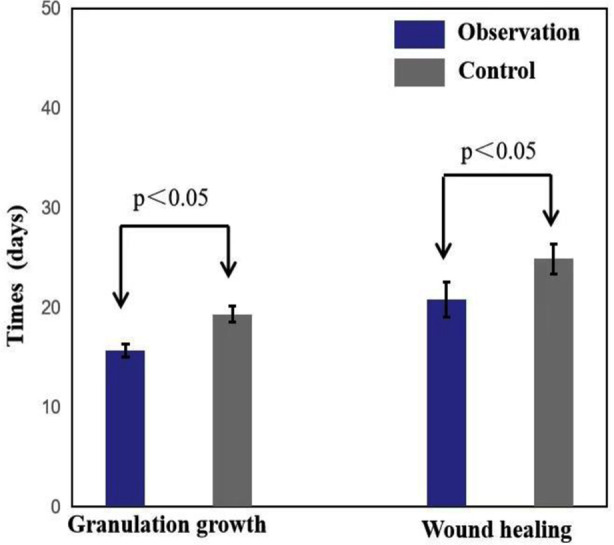
Comparison of the Granulation growth time and wound healing time between the two groups

**Fig.4:**
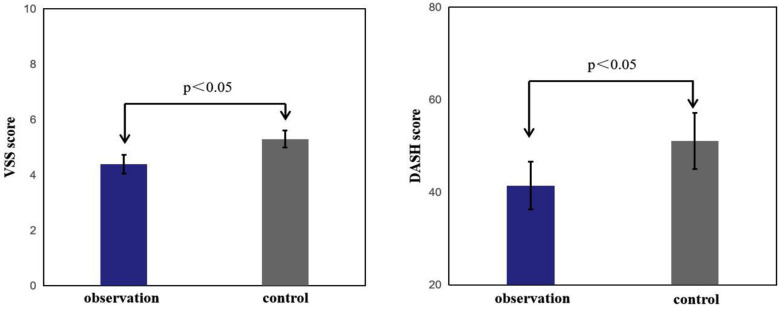
Comparison of the VSS scores and DASH scores between the two group.

The mean wound pain scores of the patients in both groups decreased over time. However, the observation group exhibited lower pain scores on days three, seven, and 14 after treatment, and the difference was significant (P<0.05). This suggests that the observation group experienced enhanced pain management (Fig.2). The observation group demonstrated shorter average times for granulation tissue growth and wound healing, and the difference was significant (P<0.05). This means that after treatment with the double-layer artificial dermis, the wound can quickly generate granulation tissue, thereby accelerating wound healing and reducing the duration of illness (Fig.3). Furthermore, the Vancouver Scar Score (VSS) and the Arm, Shoulder, and Hand Disability Score (DASH) were also lower in the observation group, and the difference was significant (P<0.05), suggesting improved functional outcomes and reduced scarring (Fig.4).

## DISCUSSION

Repairing full-thickness skin defects, particularly severe wounds with exposed tendons and bones, remains a clinical challenge. Free skin grafts or flap transplantation are therefore often employed as first-line treatment.^4^ However, the presence of other severe co-morbidities may render some patients either medically unfit or unwilling to undergo prolonged anaesthesia or flap transplantation surgery. Besides, flap surgery techniques are complex, characterized by a steep learning curve, significant surgical risks, lengthy procedures, and considerable pain. Consequently, primary hospitals may not pervasively perform such operations. Consequently, many patients opt for conventional treatments, such as dressing changes and negative pressure suction. Nevertheless, these approaches are often associated with a heightened risk of tendon necrosis, and even osteomyelitis, ultimately leading to prolonged illness and suboptimal wound healing quality.

Double-layer artificial dermis, a tissue-engineered biomaterial widely utilized in regenerative medicine, presents innovative therapeutic potential for the management of complex skin injuries. Structurally, it consists of two distinct layers: the upper layer is a non-permeable silicone membrane that serves as a temporary barrier against bacterial invasion and excessive water loss, while the lower layer is a biodegradable matrix that functions as a scaffold for cell and capillary growth. Consequently, the double layer artificial dermis has been successfully applied in the treatment of deep burns, postoperative scar excision wounds, chronic skin ulcers, and wounds after tumor excision.^15^,^16^ Therefore, we conducted this research, exploring potentially effective treatment that could replace traditional flap surgery.

According to our research, the application of double-layer artificial dermis significantly alleviates pain, particularly during dressing changes. In the observation group, a marked reduction in pain was observed as early as the second day after surgery, while patients in the control group continued to experience sustained discomfort until wound healing. Due to its double-layer structure, the upper layer functions as an antibacterial barrier, while the lower layer promotes cell growth and vascularization through its porous structure. This configuration minimizes direct irritation of the nerve endings by external stimuli, thereby alleviating pain.^15^,^17^ Some studies have suggested that the pain-relieving effect of double-layer artificial dermis is most pronounced in the early postoperative period, with no significant long-term outcomes over other methods.^18^ However, our research demonstrates that pain reduction endures from the moment of scaffold application through complete vascularization of the wound bed. We speculate that this may be attributed to the following factors. Firstly, Our study primarily addressed small-area wounds, which typically exhibit shorter vascularization time. Secondly, the most intense wound pain was caused by dressing-change irritation. As skin grafting was promptly performed following vascularization, the need for extended exposure and manipulation of the wound was minimized, thereby reducing ongoing stimulation and discomfort.

Our study revealed a markedly lower incidence of wound infection in the observation group. By contrast, the control cohort experienced three infections secondary to necrosis of the exposed tendon. Tendons in the observation group remained well-protected, exhibiting no signs of necrosis. Furthermore, patients in the control group frequently exhibited pronounced inflammatory signs - redness, swelling around the wound, and substantial exudate accumulation prior to wound healing. However, the redness and swelling in the observation group were rapidly alleviated after covering the wounds using the double-layer artificial dermis, accompanied by a marked reduction in exudate. Previous studies have indicated that its outer layer functions analogously to an epidermal barrier, actively blocking bacterial penetration.^15^ Additionally, this bilayer structure contributes to a lower incidence of secondary infections associated with frequent dressing changes.^19^ Therefore, in wounds involving exposed bone or tendons, the double-layer artificial dermis offers temporary protection, establishing optimal preparatory conditions for secondary flap or skin grafting procedures, while eliminating concerns about necrosis or infection caused by exposed bone or tendons.

Our study demonstrated that patients in the observation cohort attained significantly shorter average times to both granulation tissue growth and wound healing. The double-layer artificial dermis accelerates granulation across the wound surface, expediting the entire wound healing. In the control group, granulation typically appeared only after avascular necrosis of the exposed tendon, while the double-layer artificial dermis simultaneously shielded the tendon and provided a three-dimensional growth space for fibroblasts and vascular endothelial cells, promoting collagen deposition and angiogenesis.^15^,^20^ Consistently, the observation group recorded significantly lower VSS and DASH scores. Application of the double-layer artificial dermis accelerated granulation, curtailed the interval to secondary skin grafting and wound healing, and effectively suppressed post-healing scar hyperplasia of the hand. These advantages likely stem from its capacity to downregulate pro-inflammatory gene expression and reduce macrophage infiltration, interrupting the inflammation-induced fibrosis.^21^ By interrupting the inflammation-fibrosis vicious cycle, this approach favors true tissue regeneration over scar repair, ultimately inhibiting scar formation. Empirical evidence further shows that the bilayer scaffold raises the proportion of Type-III collagen while reducing excessive deposition of Type-I collagen.^22^ Our findings further indicate that the application of double-layer artificial dermis improved hand functionality. Owing to the relatively mild pain and minimal tissue edema at the injury site, patients exhibit greater willingness to engage in early rehabilitation exercises. Moreover, it effectively preserves tendon integrity, thereby facilitating the restoration of hand function.

Our findings indicate that, in the treatment of hand wounds with limited bone and tendon exposure, double-layer artificial dermis markedly alleviates pain, attenuates inflammatory responses, reduces infection rates, stimulates granulation tissue proliferation and wound healing, and mitigates scar hyperplasia, ultimately achieving optimal long-term functional recovery. Consequently, this method offers promising potential to revolutionize the conventional belief that flap transplantation was invariably necessary in cases where tendons or bones were exposed previously. Hence, the double-layer artificial dermis merits extensive promotion and application, particularly in primary medical institutions that possess relatively limited equipment and technical capabilities.

### Limitations:

This study specifically focuses on patients who are ineligible for skin flap transplantation or have declined this procedure. A direct comparison between artificial dermis and traditional flap surgery is not feasible within the scope of this study. Moreover, our study primarily focused on patients with extensive bone and tendon exposure areas smaller than 10cm^2^. Therefore, further research is needed to determine both: 1) The maximum permissible area of exposed bone and tendons. 2) The upper limit of wound sizes amenable to this treatment. Therefore, we will continue to pursue these inquiries in future studies to further elucidate the clinical potential of artificial dermis, including comparative analyses with skin flap transplantation.

## CONCLUSION

The double-layer artificial dermis can effectively alleviate patient pain, promote wound healing via inflammatory response suppression, control infections, and foster granulation tissue growth. Additionally, it can significantly reduce scar proliferation and enhance the hand’s aesthetic and functional recovery. Moreover, this method has the potential for widespread promotion and application in primary hospitals that are not equipped with advanced medical technology.

### Authors’ Contribution:

**CR:** Conceived, designed and did statistical analysis & editing of manuscript, is responsible for integrity of research. **JC and HO:** Did case collection, data organization. Critical review. **BX:** Critical analysis and final approval of manuscript.
